# Contemporary Mortality and Comparative Outcomes of Nephrectomy Versus Non-nephrectomy Management in Emphysematous Pyelonephritis: A Systematic Review and Meta-Analysis

**DOI:** 10.7759/cureus.106314

**Published:** 2026-04-02

**Authors:** Okelue E Okobi, Annelise Fernandez, Emasenyie Isikwei, Kadija Traoré, Vikash S Desai, Akinyele Oladimeji, Onize Ekome, Miguel Diaz-Miret, Sergio Hernandez Borges

**Affiliations:** 1 Family Medicine, Larkin Community Hospital Palm Springs Campus, Hialeah, USA; 2 Family Medicine, IMG Research Academy and Consulting LLC, Homestead, USA; 3 Family Medicine, Borinquen Medical Centers, Miami, USA; 4 Family Medicine, Obafemi Awolowo University, Ile-Ife, NGA; 5 Mental Health, Braxia Health, Mississauga, CAN

**Keywords:** emphysematous pyelonephritis, management, meta-analysis, mortality, nephrectomy, percutaneous drainage

## Abstract

Emphysematous pyelonephritis is a life-threatening necrotizing infection of the renal parenchyma characterized by gas formation and systemic toxicity. Although advances in imaging and minimally invasive techniques have altered treatment patterns, reported mortality rates and optimal management strategies remain variable across institutions. This systematic review and meta-analysis were conducted in accordance with the PRISMA guidelines to evaluate contemporary mortality and compare outcomes between non-nephrectomy management and nephrectomy. Peer-reviewed studies published between 2010 and 2025 reporting mortality in adult patients were identified through structured database searches. Quantitative synthesis was performed using R software. Mortality proportions were pooled using random-effects modeling, and ORs were calculated to compare management approaches. In total, 26 studies met the inclusion criteria for qualitative synthesis, and 11 studies comprising 523 patients were eligible for meta-analysis. The pooled mortality was 7.6% (95% CI, 5.4-10.5) under the fixed-effects model and 7.7% (95% CI, 4.4-13.3) under the random-effects model, with moderate between-study heterogeneity. Comparative analysis demonstrated significantly lower odds of mortality in patients managed without nephrectomy. These findings indicate that contemporary mortality is lower than previously reported and support a selective, stepwise management approach.

## Introduction and background

Emphysematous pyelonephritis (EPN) is a serious necrotizing infection of the upper urinary tract, involving the renal parenchyma and, in some cases, the perirenal tissues of the kidney [1]. It is most commonly seen in patients with uncontrolled diabetes mellitus and in patients with urinary tract obstruction, conditions associated with high morbidity and mortality if not promptly identified and treated [2,3]. Despite advances in imaging and critical care management, EPN remains a urological emergency because it can rapidly progress to septic shock, multi-organ failure, and death [4]. CT has improved early diagnosis; nevertheless, the optimal management strategies remain under debate [5].

Traditionally, emergent nephrectomy was considered the standard of care due to the fulminant nature of the infection and the perceived necessity to eliminate the source of infection [6]. Although nephrectomy may be lifesaving, it is associated with serious perioperative complications, particularly in patients with hemodynamic instability or multiple comorbid conditions [7,8]. Over the last 20 years, less invasive approaches, including aggressive medical management with broad-spectrum IV antibiotics and supportive care and percutaneous drainage (PCD), have become increasingly accepted [9]. These measures aim to contain the infection, preserve renal function, and minimize surgical risk [10].

The trend toward conservative and minimally invasive management has been driven by improvements in critical care, advances in interventional radiology, and enhanced risk stratification systems based on radiologic classification and clinical severity [11,12]. However, controversy remains regarding which patients can be managed with medical therapy alone, which require PCD, and which should undergo early nephrectomy [13]. Some observational studies suggest that PCD combined with antibiotics leads to lower mortality than immediate surgery, while others indicate that early nephrectomy yields similar or better outcomes in severe cases [1,10,14].

Given the rarity of EPN and the limited availability of randomized controlled trials, clinical decision-making is often guided by retrospective studies and institutional experience [15]. A comprehensive review of existing evidence is therefore necessary to clarify the relationship between treatment strategy and mortality outcomes.

The objective of this study is to evaluate the association between treatment strategy (medical management, PCD, or nephrectomy) and mortality in patients with EPN. This analysis aims to inform evidence-based clinical practice and optimize survival outcomes in this high-risk patient population by synthesizing the findings of published studies.

## Review

Methods

Eligibility Criteria and Search Strategies

This systematic review and meta-analysis was conducted in accordance with the PRISMA 2020 guidelines to ensure clarity, transparency, and reproducibility of the study process [16]. The objective was to evaluate contemporary mortality in adults with EPN and to compare outcomes between nephrectomy and non-nephrectomy management strategies. The research question was structured according to population, intervention, comparison, and outcome (PICO) components.

The population consisted of adult patients aged 18 years and older with radiologically confirmed EPN. The intervention group included patients managed without nephrectomy, defined as treatment with IV antibiotics alone or in combination with PCD. The comparison group consisted of patients who underwent surgical nephrectomy, whether performed emergently or after initial stabilization. The primary outcome of interest was all-cause mortality reported during hospitalization or short-term follow-up. When available, additional outcomes such as survival rates or treatment failure were also recorded.

A comprehensive literature search was performed using PubMed, Scopus, Web of Science, Embase, and the Cochrane Library. Studies published between January 2010 and January 2025 were considered. The search strategy combined Medical Subject Headings (MeSH) and relevant keywords, including “emphysematous pyelonephritis”, “EPN”, “medical management”, “conservative treatment”, “percutaneous drainage”, “nephrectomy”, “mortality”, “survival”, and “treatment outcome”. Boolean operators were applied so that population terms were combined with management strategies and outcome measures. Reference lists of eligible studies were also reviewed manually to identify additional relevant publications.

Table 1 provides an overview of the literature search strategy, including databases searched, time frame, and key search terms.

**Table 1:** Overview of search strategy

Category	Details
Databases searched	PubMed, Scopus, Web of Science, Embase, Cochrane Library
Time frame	Studies published between 2010 and 2025 were included
Language	Only studies published in English were considered
Search strategy structure	The search strategy was structured using a combination of three key components (#1 AND #2 AND #3)
Population (#1)	“emphysematous pyelonephritis” OR “EPN”
Intervention/comparison (#2)	“medical management” OR “conservative treatment” OR “percutaneous drainage” OR “nephrectomy”
Outcome (#3)	“mortality” OR “survival” OR “treatment outcome” OR “case fatality”

Inclusion and Exclusion Criteria

Studies were included if they enrolled adult patients with EPN and reported mortality outcomes stratified by management approach. Eligible study designs included randomized controlled trials, prospective cohort studies, retrospective cohort studies, and larger case series. Only articles published in English during the predefined study period were considered. Studies were required to provide sufficient data to extract mortality rates for quantitative synthesis.

Studies were excluded if they were case reports, conference abstracts, editorials, letters, narrative reviews, animal studies, or laboratory investigations. Articles involving pediatric populations were excluded. Studies that did not clearly specify the treatment modality or failed to report mortality outcomes were also omitted. Additionally, studies that combined EPN with other infectious conditions without presenting separate extractable data were not included in the analysis.

Screening Process

All records identified were imported into EndNote, and duplicates were removed. Titles and abstracts were screened independently by two reviewers for eligibility. Full-text articles of potentially relevant studies were then assessed independently. Discrepancies were resolved through discussion, and a third reviewer was consulted when necessary to ensure methodological rigor.

Quality Assessment

To assess the methodological quality of the articles included in the systematic review, appropriate instruments were used based on study design. Randomized trials were evaluated using the Cochrane Risk of Bias 2 (RoB 2) tool [17], and observational studies were assessed using the adapted Newcastle-Ottawa Scale (NOS) [18].

Data Extraction

A standardized data extraction form was used to collect key study characteristics, including author, year, country, study design, and sample size. Clinical variables included patient demographics, comorbid conditions (e.g., diabetes), radiologic classification of EPN, type of treatment (medical management, PCD, and nephrectomy), and mortality rates. Additional data, such as ICU admission, septic shock, and length of hospital stay, were recorded when available. Data were extracted independently by two reviewers. Discrepancies were resolved through discussion or consultation with a third reviewer to ensure accuracy and consistency across all studies.

Data Analysis

Quantitative analysis was performed to estimate pooled mortality and to compare mortality across management strategies. All statistical analyses were conducted using R version 4.5 with the meta and metafor packages [19]. Mortality proportions from individual studies were pooled using inverse variance methods after logit transformation. Both fixed-effect and random-effects models were calculated. Statistical heterogeneity was assessed using the Cochran Q test and quantified using I² and τ² statistics. The random-effects model applied the DerSimonian-Laird approach with Hartung-Knapp adjustment.

For comparative analysis, ORs were calculated to assess mortality in non-nephrectomy versus nephrectomy groups using the Mantel-Haenszel method with continuity correction when necessary.

Results

The electronic databases were searched extensively, yielding 620 articles. After removing 140 duplicates, 480 titles and abstracts were screened. Of these, 360 studies were excluded due to failure to meet the inclusion criteria, absence of stratification by treatment strategy (medical management, PCD, or nephrectomy), or lack of reported mortality outcomes. The remaining 120 full-text articles were assessed in detail. After full-text review, 94 articles were excluded due to inadequate outcome measures, uncertainty regarding the treatment group, inclusion of pediatric populations, or non-peer-reviewed materials such as conference abstracts and editorials. Ultimately, 26 articles met the inclusion criteria and were incorporated into this systematic review and meta-analysis.

The included studies were predominantly retrospective and prospective cohort studies, with a few large case series, reflecting the rarity and severity of EPN. Studies represented diverse geographic locations, including North America, Europe, Asia, and Africa, and comprised both single-center and multicenter experiences. Mortality outcomes varied according to treatment modality. Overall, PCD and medical management were associated with favorable survival compared to medical therapy alone, whereas nephrectomy was more frequently employed in severe or recurrent cases. Heterogeneity in disease severity classification and management protocols contributed to variability in the reported outcomes.

**Figure 1:**
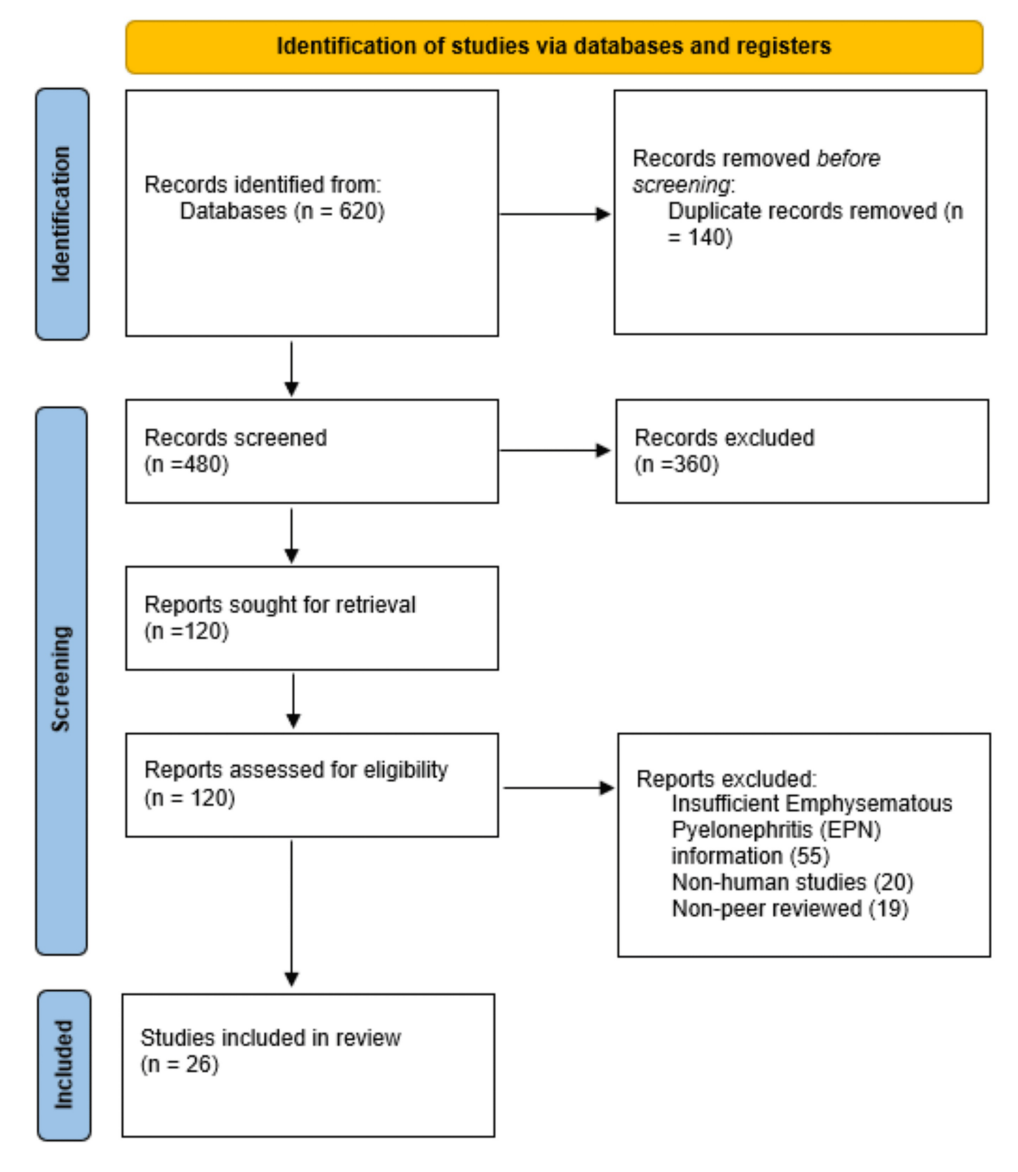
PRISMA flow diagram indicating the study selection and inclusion process This review was conducted in accordance with the PRISMA 2020 guidelines [16]. This work is licensed under CC BY 4.0.

Figure 1 illustrates the PRISMA flow diagram outlining the study selection and inclusion process.

Table 2 summarizes the characteristics of the 26 studies included in this systematic review.

**Table 2:** Summary of included studies ACS, acute coronary syndrome; EPN, emphysematous pyelonephritis; PCD, percutaneous drainage

Reference	Study design	Population	Outcome focus	Key findings
Wu et al. (2022) [1]	Review article	Patients with EPN	Classification, management, prognosis	Highlighted the importance of CT-based classification guiding management; conservative approaches increasingly favored
Sellon et al. (2013) [2]	Case report	Old-age patient with diabetes	Clinical management	EPN is prone to diabetic people
Aboumarzouk et al. (2014) [3]	Systematic review	EPN patients	Management strategies	Recommended stepwise approach prioritizing drainage before nephrectomy
Newcomer et al. (2022) [4]	Retrospective cohort	EPN patients	PCD outcomes	PCD effective in selected stable patients
Gray et al. (2021) [5]	Randomized controlled trial	ACS patients	CT coronary angiography	Not related to EPN
Aggarwal et al. (2023) [6]	Cohort study	EPN patients	Mortality and nephrectomy predictors	Shock and thrombocytopenia predicted mortality and the need for nephrectomy
Jain et al. (2019) [7]	Retrospective study	EPN patients	Algorithm-based management	Standardized protocol improved survival rates
Deoraj et al. (2018) [8]	Case report and review	EPN patients	Conservative outcomes	Conservative treatment successful in select cases
Sengupta and Basu (2021) [9]	Cohort study	EPN patients	Conservative versus minimally invasive	Minimally invasive approaches reduced mortality
Kangjam et al. (2015) [10]	Retrospective study	EPN patients	Conservative management	Conservative therapy feasible in early-stage disease
Kumar et al. (2025) [11]	Retrospective analysis	EPN patients	Clinical and radiologic profile	CT severity correlated with outcomes
Sokhal et al. (2017) [12]	Cohort study	EPN patients	Trends in management	Shift toward drainage over emergency nephrectomy
Al-Saraf et al. (2022) [13]	Observational study	EPN patients	Drainage disparities	Variability in drainage utilization influenced outcomes
Kapoor et al. (2010) [14]	Cohort study	EPN patients	Mortality predictors	Identified renal impairment and shock as mortality predictors
Rafiq et al. (2021) [15]	Prospective study	Diabetic EPN patients	Clinical outcomes	Diabetes severity linked to worse prognosis
Rahoui et al. (2022) [20]	Cohort study	EPN patients	Conservative failure predictors	Hemodynamic instability predicted failure
Alsharif et al. (2015) [21]	Retrospective study	EPN patients	Nephrectomy necessity	Nephrectomy reserved for severe or refractory cases
Rahim et al. (2021) [22]	Cohort study	EPN patients	Institutional outcomes	Multidisciplinary care improved survival
Nabi et al. (2020) [23]	Comparative study	Diabetic patients	EPN versus pyelonephritis outcomes	EPN associated with higher morbidity
Bedoui et al. (2023) [24]	Case series	EPN patients	Sepsis and mortality risk	Sepsis significantly increased mortality risk
Zhang et al. (2024) [25]	Case report	EPN patients	Treatment outcomes	Demonstrated variability in outcomes
Jiang et al. (2025) [26]	Case series and review	EPN patients	Clinical management	Reinforced role of early drainage
Gite et al. (2021) [27]	Single-center study	EPN patients	Minimally invasive techniques	First-line minimally invasive strategy improved survival
Karthikeyan et al. (2018) [28]	Retrospective study	EPN patients	Conservative outcomes	Conservative/minimally invasive treatment effective in stable cases
Kone et al. (2022) [29]	Prospective study	EPN patients	Protocol-based management	Protocol-driven care reduced mortality
Irfaan et al. (2020) [30]	Retrospective review	EPN patients	Clinical outcomes	Early intervention associated with improved survival

Table 3 presents the methodological quality assessment of the included observational studies using the NOS [18]. The results show that the majority of the observational studies were rated as high quality, with most scoring 8 or 9 points.

**Table 3:** Quality assessment for observational studies (NOS) In the NOS, the maximum score is 9 points, with 4 points for selection, 2 points for comparability, and 3 points for outcome. The NOS is a freely available tool for assessing the quality of observational studies and was applied in accordance with published guidelines [18]. NOS, Newcastle-Ottawa Scale

Study	Selection (max 4)	Comparability (max 2)	Outcome (max 3)	Total score (max 9)	Quality rating
Wu et al. (2022) [1]	4	2	3	9	High
Sellon et al. (2013) [2]	3	2	2	8	High
Aboumarzouk et al. (2014) [3]	3	2	3	8	High
Newcomer et al. (2022) [4]	4	2	3	9	High
Aggarwal et al. (2023) [6]	4	2	3	9	High
Jain et al. (2019) [7]	3	2	3	8	High
Deoraj et al. (2018) [8]	2	1	2	5	Moderate
Sengupta and Basu (2021) [9]	3	2	3	8	High
Kangjam et al. (2015) [10]	3	1	2	6	Moderate
Kumar et al. (2025) [11]	3	2	3	8	High
Sokhal et al. (2017) [12]	4	2	3	9	High
Al-Saraf et al. (2022) [13]	3	1	2	6	Moderate
Kapoor et al. (2010) [14]	4	2	3	9	High
Rafiq et al. (2021) [15]	4	2	3	9	High
Rahoui et al. (2022) [20]	4	2	3	9	High
Alsharif et al. (2015) [21]	3	1	2	6	Moderate
Rahim et al. (2021) [22]	3	1	2	6	Moderate
Nabi et al. (2020) [23]	4	2	3	9	High
Bedoui et al. (2023) [24]	4	2	3	9	High
Zhang et al. (2024) [25]	2	0	2	4	Low
Jiang et al. (2025) [26]	2	0	2	4	Low
Gite et al. (2021) [27]	4	2	3	9	High
Karthikeyan et al. (2018) [28]	4	2	3	9	High
Kone et al. (2022) [29]	4	2	3	9	High
Irfaan et al. (2020) [30]	3	1	2	6	Moderate

Table 4 shows the risk of bias assessment for the included randomized controlled trial using the Cochrane RoB 2 tool [17].

**Table 4:** Risk of bias assessment for randomized controlled trials (RoB 2 tool) The Cochrane RoB 2 tool is freely available for use and was applied according to established guidance for randomized trials [17]. RoB 2, Risk of Bias 2

Study	Randomization process	Deviations from intended interventions	Missing outcome data	Measurement of outcome	Selection of reported results	Overall risk of bias
Gray et al. (2021) [5]	Low	Low	Low	Low	Low	Low

Meta-Analysis

A total of 11 studies comprising 523 patients with EPN were included for quantitative synthesis. Across these cohorts, 33 deaths were recorded in total.

Table 5 presents the pooled mortality estimates in patients with EPN under both fixed-effect and random-effects models.

**Table 5:** Pooled mortality in EPN EPN, emphysematous pyelonephritis

Model	Mortality %	95% CI	I²	τ²	p Heterogeneity
Fixed effect	7.6	5.4-10.5	-	-	-
Random effects	7.7	4.4-13.3	41.7%	0.274	0.071

**Figure 2:**
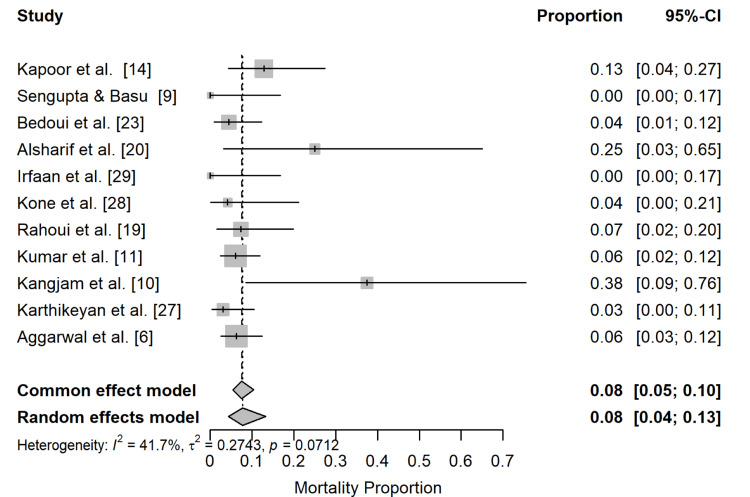
Pooled mortality proportion forest plot

The findings show that the pooled mortality was 7.6% under the fixed-effects model compared to 7.7% in the random-effects model. The reported CIs were 5.4-10.5 and 4.4-13.3, respectively. Given the I² value of 41.7%, there is moderate heterogeneity, with a nonsignificant heterogeneity test (p = 0.07 > 0.05). These findings are illustrated in Figure 2, which presents the pooled mortality proportion across the 11 included studies in the meta-analysis.

The figure shows an observed mortality range of 0-37.5%, reflecting the lowest and highest study-specific estimates included in the meta-analysis. Several cohorts reported no in-hospital deaths, resulting in a mortality proportion of 0%.

Table 6 shows the comparative meta-analysis evaluating mortality by management strategy.

**Table 6:** Random effects meta-analysis of mortality comparing non-nephrectomy management versus nephrectomy

Model	OR	95% CI	I²	τ²	p Heterogeneity
Fixed effect	0.12	0.05-0.33	-	-	-
Random effects	0.12	0.05-0.27	0.0%	0.00	0.877

**Figure 3:**
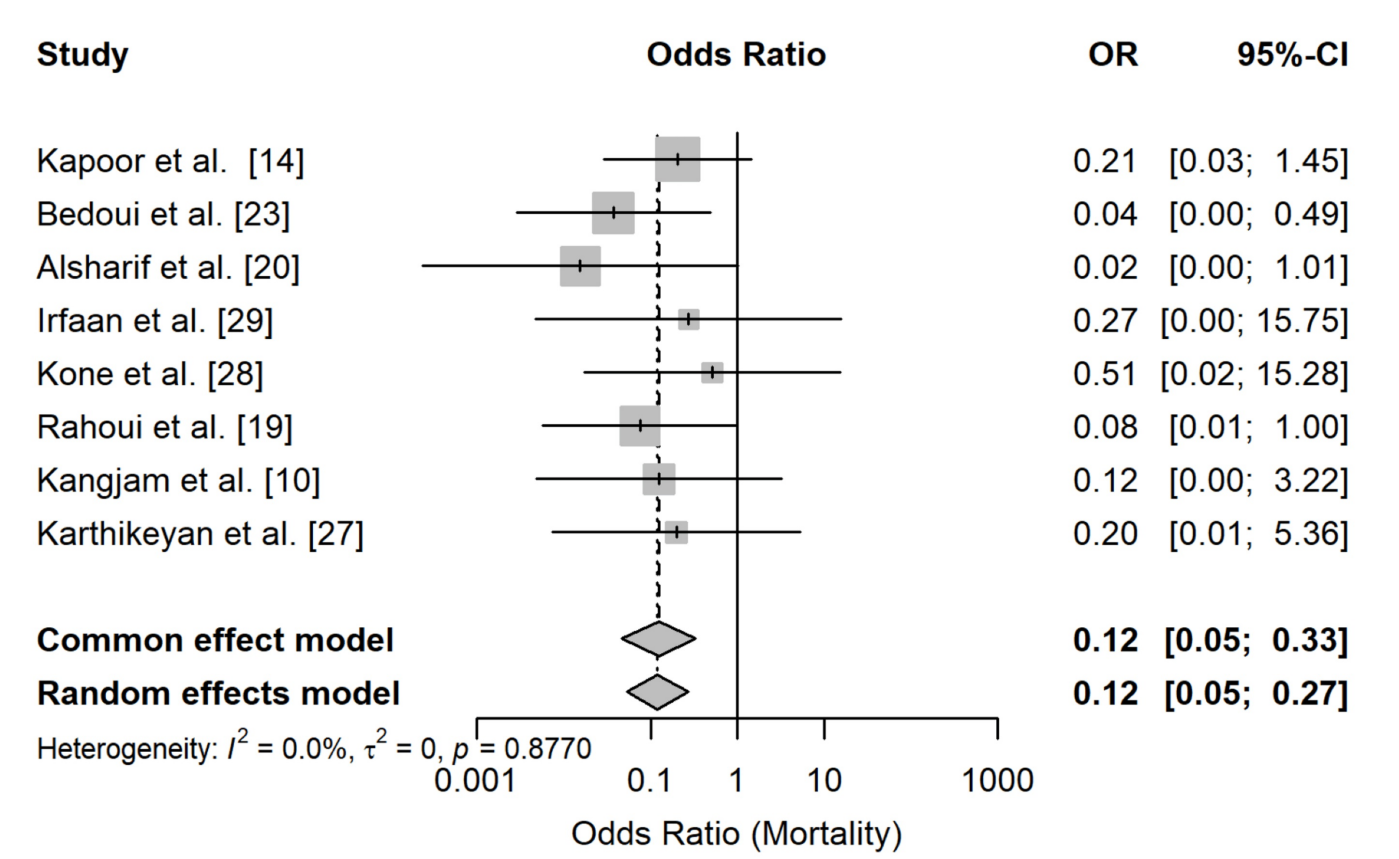
Forest plot of pooled ORs for mortality comparing non-nephrectomy management versus nephrectomy in EPN EPN, emphysematous pyelonephritis

The results revealed that non-nephrectomy management was associated with lower odds of mortality compared to nephrectomy. The pooled OR under the random-effects model was 0.12 (95% CI, 0.05-0.27), indicating statistical significance, as the 95% CI did not include 1. As shown in Figure 3, which presents the pooled OR for mortality comparing non-nephrectomy management with nephrectomy, individual study estimates consistently favored non-nephrectomy management.

Study Findings

This systematic review has shown that conservative and minimally invasive approaches are increasingly associated with greater survival in patients with EPN compared to routine early nephrectomy, especially in hemodynamically stable patients [1,3]. In the cohort and prospective studies included, mortality was closely related to shock, thrombocytopenia, kidney failure, and increased radiologic class rather than the treatment modality itself [6,20,21].

Various studies demonstrated the significance of CT-based categorization systems in guiding individualized treatment decisions. Wu et al. [1] and Kumar et al. [11] demonstrated that radiologic severity was closely associated with prognosis and the need for escalation to more invasive interventions. Similarly, Jain et al. [7] showed that a standardized management algorithm incorporating clinical severity optimized patient outcomes and minimized mortality.

Retrospective and prospective cohort evidence has demonstrated that PCD combined with broad-spectrum IV antibiotics yields favorable survival outcomes, particularly in patients without refractory shock. Newcomer et al. [4] also identified clinical predictors of successful nonoperative management, while Sengupta and Basu [9] reported lower mortality rates when minimally invasive strategies were used as first-line approaches. Sokhal et al. [12] validated the international trend of replacing emergency nephrectomy with a drainage-based approach, a shift facilitated by the development of interventional radiology and enhanced critical care. Additionally, Rahim et al. [22] affirmed that early identification, ICU-based monitoring, and prompt intervention improved survival even in high-risk patient groups.

In conclusion, nephrectomy remains necessary in severe or refractory cases; however, with appropriate risk stratification based on radiologic classification and clinical severity markers, conservative or minimally invasive interventions can be safely implemented [6,14,21]. These approaches are associated with better renal preservation and similar or even lower mortality rates in properly selected patients with EPN.

Discussion

In this study, the meta-analysis reveals that mortality in EPN is lower than historically reported rates. Across the 11 eligible studies, which included 523 patients, the pooled mortality was 7.7% under the random-effects model. Between-study heterogeneity was moderate, indicating some clinical variability across cohorts. Furthermore, the comparative analysis revealed a significant difference in mortality according to management strategy. Non-nephrectomy approaches were associated with lower odds of death compared with nephrectomy.

Conservative (Medical) Management and Mortality Outcomes

Broad-spectrum antibiotics, glycemic control, hemodynamic stabilization, and supportive care comprise conservative management, which has gradually been considered a first-line treatment option in selected patients with EPN. Recent research indicates that early diagnosis and aggressive medical treatment may lead to acceptable survival rates, especially in hemodynamically stable patients who have not experienced extensive parenchymal damage [1,12]. Several retrospective studies have shown that carefully selected patients treated conservatively achieved favorable outcomes without undergoing emergency nephrectomy [9,10].

Risk stratification is important for determining the appropriateness of medical therapy. Predictors of treatment failure include thrombocytopenia, altered mental status, shock, acute kidney injury, and extensive radiologic involvement [20,23,24]. Studies on populations with diabetes highlight the importance of tight metabolic control, given the strong correlation between EPN and poorly controlled diabetes [15,23]. Case series and institutional experiences also demonstrate that early intervention in unstable patients may increase mortality, emphasizing the need to identify high-risk features promptly [22,25,26]. Overall, conservative management appears safe in appropriately selected low-risk patients but requires strict monitoring and escalation if deterioration occurs.

Minimally Invasive Approaches (PCD) and Clinical Outcomes

PCD has become a cornerstone of EPN treatment, serving as an intermediate strategy between conservative therapy and radical surgery. Image-guided drainage combined with antibiotics has been suggested to improve infection control while preserving renal function [4,27,28]. Comparative studies have shown that PCD lowers mortality compared to immediate nephrectomy, particularly in patients with localized disease or those who are inoperable [7,9].

Predictors of successful PCD include limited gas extension, absence of multi-organ failure, and early intervention [4,20]. Management algorithms incorporating PCD as first-line therapy have demonstrated improved survival and reduced need for nephrectomy [7,29]. However, accessibility to interventional radiology may influence treatment choice and outcomes across different healthcare settings [13]. Overall, minimally invasive strategies provide a reasonable compromise, reducing surgical morbidity without compromising survival [30].

Surgical Management (Nephrectomy), Risk Stratification, and Mortality Predictors

Emergency nephrectomy was traditionally considered the optimal treatment for EPN despite high mortality rates, with past research emphasizing aggressive surgical debridement to control sepsis [3,14]. More recent studies recommend nephrectomy only in cases of widespread renal damage or sepsis unresponsive to conservative and minimally invasive therapies [1,21].

Factors such as hemodynamic instability, septic shock, thrombocytopenia, and delayed presentation are strongly associated with mortality after nephrectomy [6,24]. Risk-based scoring and classification models have been proposed to guide decision-making and predict outcomes [7,12]. The current evidence supports an individualized approach: surgery remains lifesaving in severe cases but is no longer universally necessary. This represents a shift toward risk-modulated, stepwise management to maximize survival and preserve renal function whenever possible.

Limitations

Several limitations of this study should be acknowledged. First, the majority of the included studies were small cohorts, retrospective, and single center, which restricted the ability to generalize findings and incorporate a larger number of studies. Second, there was considerable heterogeneity in disease severity classification, treatment protocols, and outcome reporting, which limited direct comparisons. Third, only studies published between 2010 and 2025 were included, potentially excluding relevant studies published before 2010. Additionally, the findings and overall pooled estimates may have been affected by publication bias and the exclusion of non-English studies.

## Conclusions

This systematic review and meta-analysis indicate that mortality in EPN is lower than previously reported. Comparative analysis shows that non-nephrectomy management, including conservative therapy and PCD, is associated with lower mortality than nephrectomy. These findings support an individualized approach guided by clinical stability and radiologic severity. PCD serves as an effective intermediate strategy in appropriately selected patients, while nephrectomy remains necessary in severe or refractory cases. Early risk assessment and a structured stepwise treatment approach are important for improving survival. Further prospective multicenter studies are needed to standardize management and refine outcome prediction.
